# Oncogenic Human Papillomaviruses Drive One‐Third of Sinonasal Squamous Cell Carcinoma and Are Not Mutually Exclusive for Gene Mutations

**DOI:** 10.1002/hed.28084

**Published:** 2025-01-25

**Authors:** Maxime Henrion, Valérie Costes‐Martineau, Ignacio González Bravo, Nathalie Boulle, Jérôme Solassol, Julie Vendrell, Renaud Garrel, Aude Trinquet, Vanessa Lacheretz‐Szablewski

**Affiliations:** ^1^ Departement de Pathologie Centre Hospitalo‐Universitaire Montpellier Montpellier France; ^2^ Laboratoire MIVEGEC, Centre National de la recherche Scientifique (CNRS) Montpellier France; ^3^ Laboratoire de Biologie des Tumeurs Solides, Centre Hospitalo‐Universitaire Montpellier Montpellier France; ^4^ Departement de chirurgie cervico faciale, cancérologie et laryngologie Centre Hospitalo‐Universitaire Montpellier Montpellier France

**Keywords:** genotype, in situ hybridization, mutations, oncogenic human papillomaviruses, sinonasal squamous cell carcinoma

## Abstract

**Background:**

The detection rate of oncogenic human papillomaviruses (HPVs) in sinonasal squamous cell carcinomas (SNSCCs) varies among studies. The mutational landscape of SNSCCs remains poorly investigated.

**Methods:**

We investigated the prevalence and prognostic significance of HPV infections based on p16 protein expression, HPV‐DNA detection, and E6/E7 mRNA expression using immunohistochemistry, polymerase chain reaction, and in situ hybridization, respectively. In addition, we evaluated the genetic mutations in 59 patients using next‐generation sequencing.

**Results:**

One‐third of the SNSCCs were truly oncogenic HPV‐driven tumors associated with a nonkeratinizing morphology (*p* = 0.01) and did not correlate with the prognosis. The following gene mutations were detected: *TP53*, *PIK3CA*, *CDKN2A*, *EGFR*, and *FGFR3*. These mutations occurred alone, in association with, or with oncogenic HPV.

**Conclusion:**

One‐third of SNSCCs were high‐risk HPV driven lesions. However, gene mutations and HR‐HPV infections are not mutually exclusive. Further studies are required to analyze the prognostic value of these associations.

## 1 Introduction

Squamous cell carcinoma (SCC) is the most common malignant tumor of the sinonasal tract followed by adenocarcinoma [1, 2, 3]. The prognosis of SCC is poor, with a 5‐year survival rate of 35% [3]. Tobacco use, industrial exposure, and malignant transformation from inverted sinonasal papilloma (ISP) or oncocytic sinonasal papilloma (OSP) have been reported as risk factors of sinonasal SCC (SNSCC) [4, 5, 6].

At the molecular level, chronic infection with oncogenic human papillomaviruses (HPVs) is likely the etiological factor for a significant proportion of head and neck SCCs (HNSCCs) [7]. According to the International Agency for Research on Cancer (IARC), oncogenic HPVs, also known as high‐risk HPVs (HR‐HPVs), have shown sufficient evidence of carcinogenicity in humans and experimental animals, and they include HPV types 16, 18, 31, 33, 35, 39, 45, 51, 52, 56, 58, and 59. Nononcogenic HPVs or low‐risk HPVs (LR‐HPVs) have demonstrated inadequate evidence of carcinogenicity in humans and experimental animals, corresponding to HPV types 6 and 11 [8]. The presence of oncogenic HPVs has been reported in 8.9%–30% of the SNSCC cases [9, 10, 11, 12, 13]. Discrepancies in the burden of HPV‐related SNSCCs can be explained by the techniques and criteria used to determine the viral etiology. The detection of viral DNA in cancer cells alone does not provide sufficient evidence for a causal link between infection and cancer because this approach does not differentiate between transient, silent, chronic, and oncogenic active infections. Notably, some authors do not consider HNSCC to be oncogenic HPV‐driven unless it is positive for viral DNA, as well as for the combination of viral *E6/E7* mRNA and/or cellular p16 overexpression [7, 14, 15]. Mutations in the epidermal growth factor receptor (*EGFR*) have been identified in ISPs and ISPs associated with SCC (ISP‐SCC) [12, 13, 16, 17], whereas V‐ki‐ras2 Kirsten rat sarcoma viral oncogene homolog (*KRAS*) mutations have been reported in OSPs and OSPs associated with SCC (OSP‐SCC) [18]. Mutations in other loci have also been reported albeit in small series of ISPs and ISP‐SCCs, including cyclin‐dependent kinase inhibitor 2A (*CDKN2A*), lysine methyltransferase 2D (*KMT2D*), neurofibromin 1 (*NF1*), phosphodiesterase 4D interacting protein (*PDE4DIP*), cytochrome P450 family 2 subfamily D member 6 (*CYP2D6*), fms‐related receptor tyrosine kinase 4 (*FLT4*), tumor protein p53 (*TP53*), and myosin heavy chain 9 (*MYH9*) [19].

In the present study, we determined the rate and clinicopathological significance of transcriptionally active oncogenic HPV infections in SNSCCs based on p16 protein expression and HPV‐DNA detection using immunohistochemistry (IHC) and polymerase chain reaction (PCR), respectively. Discordant cases were further analyzed using in situ hybridization (ISH) for evaluation of *E6*/*E7* mRNA expression. In addition, we analyzed Rb protein expression since p16 overexpression and loss of Rb expression have been reported to correlate with oncogenic HPV‐driven infections in oropharyngeal SCC (OPSCC) [20]. Furthermore, we evaluated the gene mutations potentially involved in the development of SNSCCs using next‐generation sequencing (NGS).

## 2 Materials and Methods

### 2.1 Case Selection

We retrospectively collected biopsy and/or surgically resected specimens of formalin‐fixed paraffin‐embedded (FFPE) tissues from 59 patients with SNSCC from the Department of Pathology, Centre Hospital Universitaire (CHU), Montpellier, France, between 1996 and 2021. For IHC and molecular analyses, we excluded specimens collected after chemotherapy or radiotherapy, as well as specimens, for which decalcification had been performed since this process may lead to protein and molecular acid degradation. The clinical information of the patients was obtained from their medical records. This study was approved by the Institutional Review Board of CHU Montpellier (No. 202201172).

### 2.2 Histological Evaluation

Four pathologists (V.L.‐S., V.C.‐M., A.T., and M. H.) reviewed and made the diagnoses according to the World Health Organization (WHO) classification of the Pathology and Genetics of Head and Neck Tumors [21] and staged the tumors according to the seventh edition of the UICC TNM classification. The cases were histologically classified into two categories: keratinizing SCC (KSCC) or nonkeratinizing SCC (NKSCC). Preexisting and/or concomitant components of ISP or OSP were evaluated.

### 2.3 p16 and Rb Immunohistochemistry

IHC was performed using 4‐μm thick FFPE tissue sections and primary monoclonal antibodies for p16 (E6H4, prediluted, CIN Histology Kit; Roche, Heidelberg, Germany) and Rb (RB1, OTI3F11, dilution 100×; LS BIO, Shirley, Massachusetts, USA), according to the manufacturers' protocols.

Staining was performed by all four study pathologists. p16 was considered positive if there was at least 70% nuclear and cytoplasmic expression with strong intensity [22]. The Rb protein expression status was classified, as follows: complete loss (CL) if there was nuclear expression in < 10% (disappeared in > 90%) of the tumor cells; preserved expression if there was nuclear Rb expression in > 90% of the tumor cells; and partial loss (PL) if there was nuclear Rb expression in ≥ 10% to ≤ 90% of the tumor cells [12, 20].

### 2.4 FFPE Sample Preparation and Nucleic Acid Extraction

FFPE tissue samples were sectioned for histological and DNA analyses. For DNA extraction, 10 consecutive 10 μm sections were transferred using a sterile wooden stick into two 1.5‐mL Eppendorf tubes. FFPE blocks were processed under strict pre−/post‐PCR conditions (physical separation), and blank paraffin blocks were systematically tested in parallel to serve as sentinels for contamination.

DNA was extracted using the Maxwell RSC DNA FFPE kit (Promega, Madison, WI, USA), according to the manufacturer's recommendation. Extracted DNA was quantified using the Qubit dsDNA high sensitivity assay kit and a Qubit fluorometer (Thermo Fisher Scientific, Waltham, MA, USA), and amplification of the gDNA extracted from the FFPE samples was determined by quantitative PCR (qPCR) using the KAPA SYBR FAST Master Mix Universal (Kapa Biosystems, Wilmington, USA), as previously described [23]. Poor‐quality gDNA not suitable for NGS analysis was excluded from the study.

### 2.5 HPV‐DNA Detection and Genotyping

HPV genotyping was performed in duplicate using 10 μL of DNA and an INNO‐LiPA HPV Genotyping *Extra II* assay kit (Fujirebio, Les Ulis, France). This test, based on reverse hybridization after a PCR step, allows the type‐specific detection of 32 viral genotypes within the *Alphapapillomavirus* genus, including oncogenic HPVs (HPV16, 18, 31, 33, 35, 39, 45, 51, 52, 56, 58, and 59), possibly oncogenic HPV (HPV68), probably oncogenic HPVs (HPV26, 53, 66, 67, 70, 73, and 82), and nononcogenic/unclassified HPVs (HPV6, 11, 40, 42, 43, 44, 54, 61, 62, 81, 83, and 89).

### 2.6 NGS Analysis

Libraries were prepared following the manufacturers' procedures using approaches specifically developed for FFPE samples (TruSeq Custom Amplicon protocol [Illumina, Evry, France] and Advanta Solid Tumor NGS Library Prep Assay with the automated Juno system [Fluidigm, San Francisco, CA, USA]). The panels allowed the detection of somatic alterations in 35 and 33 oncology‐relevant genes, respectively (Table S1). For each protocol, two libraries were prepared for each sample to minimize fixation‐related DNA damage, which could induce base modifications and generate artifacts. After preparation, the libraries were quantified, normalized, and paired‐end sequenced using a NextSeq instrument (2 × 150 cycles, Illumina). After sequencing, the four FastQ files generated per sample were automatically analyzed using a bioinformatics workflow as previously described [24]. The reported variants were annotated according to the recommendations of the American College of Medical Genetics (ACMG). Only variants reported to be pathogenic, likely pathogenic, or of unknown significance have been reported. Otherwise, the sample was designated as wild‐type (WT).

### 2.7 HPV In Situ Hybridization

We performed ISH to detect *E6/E7* mRNAs transcribed from HPV16, HPV18, and HPV33 using genotype‐specific probe sets (Ventana Medical Systems, Tucson, Arizona, USA), according to the manufacturer's protocol. Nuclear or cytoplasmic dots were considered positive [11].

### 2.8 Statistical Analysis

Statistical tests were performed using GraphPad Prism 10.0 (GraphPad). Fisher's exact test was used to evaluate associations between different markers. Overall survival (OS) and relapse‐free survival (RFS) rates were calculated using the Kaplan–Meier method. A *p* value < 0.05 was considered statistically significant.

## 3 Results

### 3.1 Clinicopathological Finding

The clinicopathological characteristics of the 59 patients are summarized in Table 1. Most patients were males (*n* = 42, 71%), and the tumors were located predominantly in the sinus (*n* = 31, 52.5%). Low stage (T1/T2 N0) and high stage (T3/T4 or N+) were similar (*n* = 29, 49% and *n* = 30, 51%, respectively). Histologically 29 (49%) cases were KSCC and 30 (51%) were NKSCC. Sixteen patients (27%) had SCC associated with IPS. For initial therapy, 20 patients (33.9%) underwent surgery alone, seven patients (11.8%) were treated with chemoradiation, 31 patients (52.5%) underwent surgery and chemoradiation, and one patient (0.17%) received no therapy. Twenty‐three patients (40%) experienced a relapse or disease progression. In total, 33 (56%) patients died because of their tumors.

**TABLE 1:** Clinicopathologic characteristics of the 59 cases of SNSCC.

	All (*N* = 59), *n* (%)
Age (y)	
Mean (range)	61.9 (53–68)
Sex	
Male	42 (71)
Female	17 (29)
Tumor site	
Sinus	31 (52.5)
Nasal cavity	28 (47.5)
TN stage	
T1/T2 N0	29 (49)
T2/T3 and/or N+	30 (51)
Tumor differentiation	
KSCC	29 (49)
NKSCC	30 (51)
Associated papilloma	
Yes	16 (27)
No	43 (73)
Relapse or progression after initial therapy	
Yes	23 (40)
No	36 (60)
Died of disease	
Yes	33 (56)
No	26 (44)

Abbreviations: KSCC: keratinasing squamous cell carcinoma; NKSCC: nonkeratinasing squamous cell carcinoma.

### 3.2 HPV Infection

**FIGURE 1:**
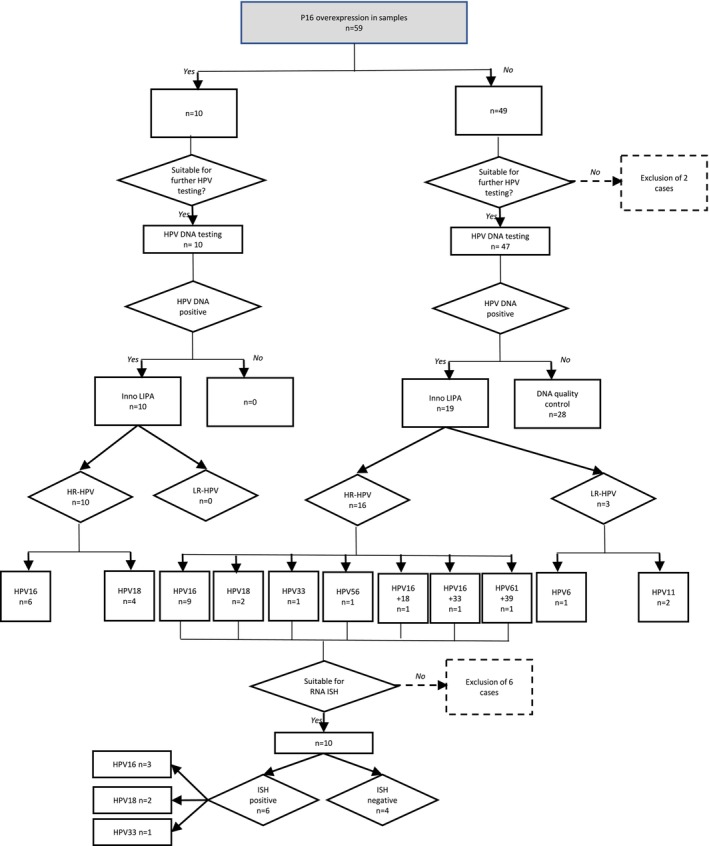
Study algorithm for the detection of p16 overexpression, HPV‐DNA, and *E6*/*E7* mRNA by polymerase chain reaction (PCR), HPV genotyping, and in situ hybridization (ISH), respectively. HR‐HPV, high‐risk HPV or oncogenic HPV; LR‐HPV, low‐risk HPV or nononcogenic HPV. [Color figure can be viewed at wileyonlinelibrary.com]

Figure 1 shows the results of p16 protein expression, HPV‐DNA detection, and genotyping.

After pathological evaluation, 57 samples were tested for HPV‐DNA, and all yielded valid DNA results. HPV‐DNA was detected in 29 (29/57, 50.1%) samples. Repartition of HPV genotypes in these 29 samples was as follows: 15 HPV16 (15/29, 51.7%), six HPV18 (6/29, 20.7%), one HPV33 (1/29, 3.4%), one HPV56 (1/29, 3.4%), one HPV16 + 18 (1/29, 3.4%), one HP16 + 33 (1/29, 3.4%), one HPV61 + 39 (1/29, 3.4%), two HPV11 (2/29, 6.9%), one HPV6 (1/29, 3.4%).

All 59 samples were tested for p16 overexpression. Ten tumors (10/59, 17%) showed p16 overexpression. These 10 samples could be tested for HPV‐DNA, genotyping, and all of them tested positive for oncogenic HPV‐DNA. Among the tumors without p16 protein overexpression (49/59, 83%), 47 were tested for HPV‐DNA and genotyped after histological evaluation. Nineteen patients (19/47, 41.3%) tested positive for HPV‐DNA. HPV genotyping revealed three LR‐HPV, two HPV11, and one HPV6.

**FIGURE 2:**
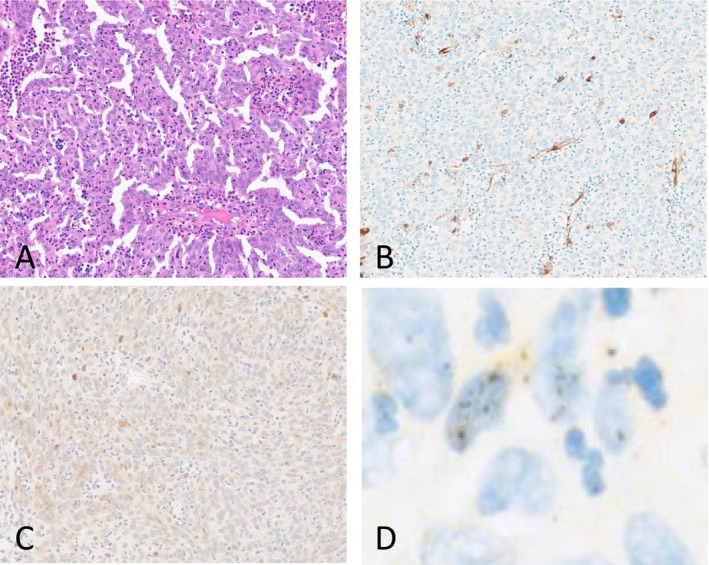
A discordant case: p16 negative/oncogenic HPV positive with a complete loss of Rb expression and positive ISH result. (A) Hematoxylin and Eosin 10×: Non keratinizing squamous cell carcinoma; (B) p16 expression 20×: No p16 overexpression; (C) Rb expression 20×: Complete loss of expression; and (D) ISH for *E6*/*E7* mRNA 60×: Nuclear and cytoplasmic dots indicating oncogenic HPV *mRNA*. [Color figure can be viewed at wileyonlinelibrary.com]

Samples that were p16 negative but with oncogenic HPV‐DNA were considered as discordant cases. These discordant cases were further investigated by ISH for *E6/E7* mRNA. We used *E6/E7* mRNA probe set for three types of oncogenic HPVs (HPV16, 18, and 33); only positive cases for these genotypes after HPV genotyping were tested (14 cases). Four patients were excluded from this study. Of the remaining 10 cases, six and four cases were ISH positive and negative, respectively. Figure 2 shows a discordant p16 negative, HPV‐DNA positive, and *E6/E7* mRNA‐positive case.

Finally, the fraction of cases attributable to oncogenic HPV was based on the positivity for HPV‐DNA and either p16 overexpression or RNA‐ISH positivity. After excluding LR‐HPV cases (three lesions) and nonevaluable cases (eight lesions), we concluded that 16 (16/48, 33.3%) were oncogenic HPV‐driven tumors. The genotypes of these 16 cases were as follows: nine HPV16 (56.25%), six HPV18 (37.5%), and one HPV33 (6.25%).

### 3.3 Rb Expression and HPV Infection

Thirty‐nine cases were analyzed for Rb protein expression. Nineteen (19/39, 48.7%) had preserved Rb expression (Rb protein expression was detected in > 90% of the carcinoma cells). Among these cases, 17 were attributable to oncogenic HPV infection. Of those, two cases (2/17, 11.8%) had oncogenic HPV‐driven lesions (one HPV16 and one HPV18). Seven cases (7/39, 17.9%) showed a complete loss of Rb expression (Rb protein expression was detected in < 10% of the carcinoma cells). All of these cases (7/7, 100%) were oncogenic HPV‐driven tumors (three HPV16 and four HPV18). Thirteen cases (13/39, 33.3%) showed a partial loss of Rb expression (Rb expression was present in ≥ 10% to ≤ 90% of the tumor cells). Among these cases, 10 were attributable to oncogenic HPV infection. Four (4/10, 40%) were oncogenic HPV‐driven lesions (two HPV16, one HPV18, and one HPV33). Most oncogenic HPV‐driven lesions (11/13, 84.6%) showed a complete or partial loss of Rb expression. Figure 2 shows an oncogenic HPV‐driven lesion with a complete loss of Rb expression but without p16 overexpression. The sensitivity of the use of complete or partial loss of Rb expression to predict active infections was 84.6% (11/13), whereas the specificity was 71.4% (15/21), predictive positive value (PPV) was 64.7% (11/17), and predictive negative value (PNV) was 88.2% (15/17). In contrast, the sensitivity of the use of p16 overexpression to predict active infections was 62.5% (10/16), the specificity was 100% (32/32), the PPV was 100% (10/10), and the PNV was 84.2% (32/38). When p16 overexpression was combined with a complete or partial loss of Rb expression to predict active oncogenic HPV infections, the sensitivity was 53.8% (7/13), specificity was 100% (20/20), PPV was 100% (7/7), and PNV was 77% (20/26).

Finally, five cases with all interpretable results (p16 and Rb expression and HR‐HPV infection status) were considered discordant cases with negative p16 expression but with a positive oncogenic HPV active infection. Among these cases, three (3/5, 60%), one (1/5, 20%), and one (1/5, 20%) had partially lost, completely lost, and preserved Rb expression, respectively.

### 3.4 Human Mutational Landscape in Sinonasal Squamous Cell Carcinomas

**FIGURE 3:**
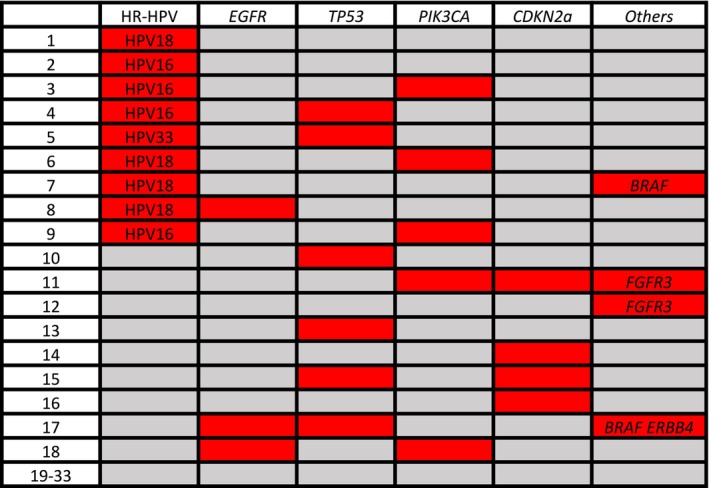
The clustering data analysis of the presence of oncogenic HPV active infection and gene mutations. Red and gray indicate positive and negative results for each factor, respectively. [Color figure can be viewed at wileyonlinelibrary.com]

Among the 59 SNSCC cases included in the study, gDNA was correctly analyzed for the mutational landscape in 33 cases (Figure 3). Mutations at the targeted loci were detected in 17 patients. *TP53* mutations were present in seven cases, either isolated (*n* = 2), in association with mutations at other loci (*EGFR* mutations, *n* = 1; *CDKN2a* mutations, *n* = 1), or with oncogenic HPVs (HPV16, *n* = 2; HPV33, *n* = 1). *PIK3CA* mutations were present in five cases, as follows: isolated (*n* = 1), *CDKN2a* mutations (*n* = 1), and oncogenic HPVs (HPV16, *n* = 2; HPV18, *n* = 1). *CDKN2a* mutations were observed in four tumors, either isolated (*n* = 1) or associated with other mutations (*PIK3CA*, *n* = 1; *EGFR*, *n* = 1; and *TP53*, *n* = 1). *EGFR* mutations were detected in three of the 33 tumors (9.1%). *EGFR* mutations were detected only in ISP‐SCCs (3/8, 37.5%), and one tumor was associated with HPV18. *BRAF* mutations were present in two cases, either associated with HR‐HPV (HPV 18) or *EGFR*, *TP53*, and *ERBB4* mutations. *FGFR3* mutations were present in two cases, either isolated or associated with *PIK3CA* and *CDKN2a* mutations.

**FIGURE 4:**
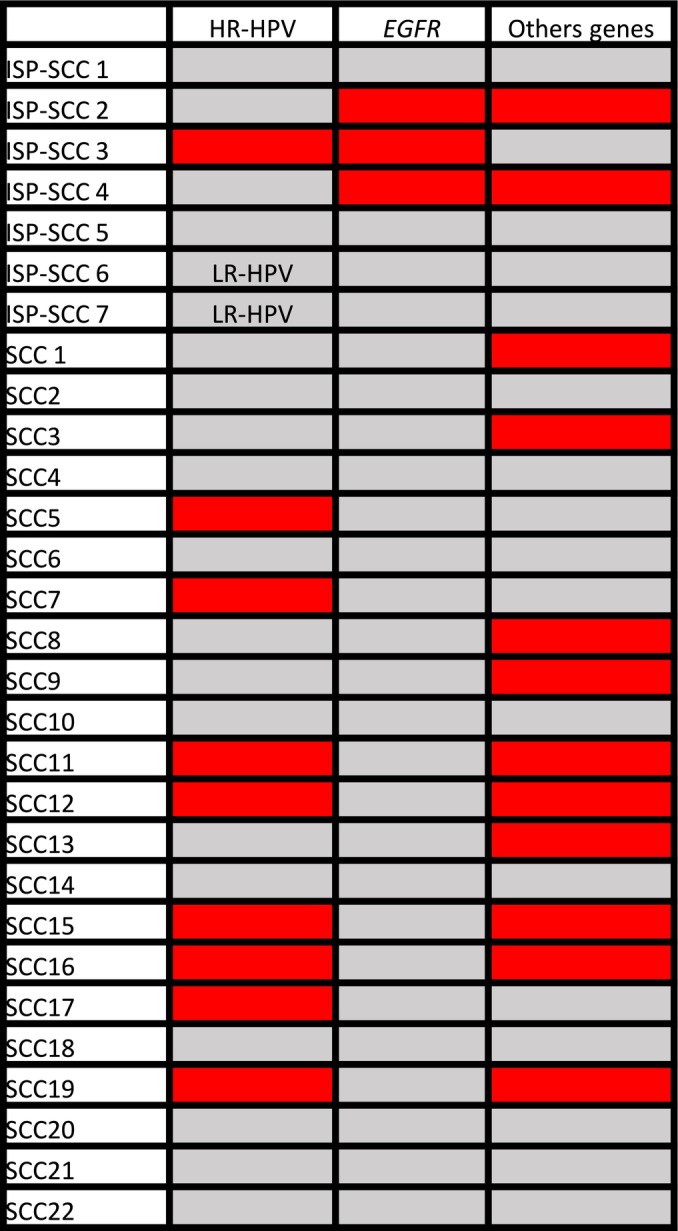
Data analysis of the presence of inverted sinonasal carcinoma, HPV infection, *EGFR* mutations, and other gene mutations. Red and gray indicate positive and negative results for each factor, respectively. ISP‐SCC, inverted sinonasal papilloma‐squamous cell carcinoma; LR‐HPV, low‐risk human papillomavirus; SCC, squamous cell carcinoma. [Color figure can be viewed at wileyonlinelibrary.com]

Figure 4 shows the data analysis of the presence of ISP components, HPV infection, and gene mutations. All data were available for 29 patients (seven ISP‐SCCs and 22 de novo SCCs). *EGFR* mutations were present only in the ISP‐SCC cases (3/7), in association with other gene mutations (two cases) or HR‐HPV (one case). Furthermore, we identified LR‐HPV in two cases of ISP‐SCCs. Oncogenic HPV infection was present in eight cases of SCCs (8/22), either isolated (3/8) or in association with gene mutations other than *EGFR* (5/8).

### 3.5 Association Between the Clinicopathological Variables, Genes Mutations, and Oncogenic HPV Active Infection

The relationships between active oncogenic HPV infection and clinicopathological variables are summarized in Table 2. Oncogenic HPV‐driven lesions were significantly associated with nonkeratinizing morphology (*p* = 0.01). Other variables tested (age, sex, tumor site, stage, associated papilloma, relapse or progression after initial therapy, and death from disease) did not show a significant association with the oncogenic HPV status.

**TABLE 2:** Association between clinicopathologic variables and HR‐HPV infection in 48 cases of SNSCC.

Variables	Total *N* = 48	HR‐HPV infection, *n* (%)		*p*
		Positive	Negative	
		16 (33.3)	32 (66.7)	
Age (y)				
≤ 60	16	6 (37,5)	10 (62,5)	0.75
> 60	32	10 (31.3)	22 (68.7)	
Sex				
Male	34	11 (32.4)	23 (67.6)	1.00
Female	14	5 (35.7)	9 (64.3)	
Tumor site				
Sinus	26	7 (26.9)	19 (73.1)	0.37
Nasal cavity	22	9 (41)	13 (59)	
T N stage				
T1/T2 N0	23	7 (30.4)	16 (69.6)	0.76
T3/T4 and/or N+	25	9 (36)	16 (64)	
Tumor differentiation				
KSCC	25	4 (16)	21 (84)	0.01
NKSCC	23	12 (52.2)	11 (47.8)	
Associated papilloma				
Yes	10	3 (30)	7 (70)	1.00
No	38	13 (34.2)	25 (65.8)	
Relapse or progression after initial therapy				
Yes	18	4 (22.2)	14 (77.8)	0.34
No	30	12 (40)	18 (60)	
Died of disease				
Yes	23	9 (39.1)	14 (60.9)	0.54
No	25	7 (28)	18 (72)	

Abbreviations: KSCC: keratinasing squamous cell carcinoma; NKSCC: NONkeratinasing squamous cell carcinoma.

Table 3 shows the association between gene mutations and active oncogenic HPV infection in 29 cases with all available information. Interestingly, gene mutations were significantly less common in nononcogenic HPV‐driven lesions than in oncogenic HPV‐driven lesions (*p* = 0.05). Nevertheless, when focusing on individual mutations, we did not identify any differences between nononcogenic HPV‐ and oncogenic HPV‐driven lesions.

**TABLE 3:** Association between genes mutations and HR‐HPV infection in 29 cases of SNSCC.

Variables	Total *N* = 29	HR‐HPV infection, *n* (%)		*p*
		Positive	Negative	
		9 (31)	20 (69)	
Genes mutations				
Yes	14	7 (50)	7 (50)	0.05
No	15	2 (13.3)	13 (86.6)	
*EGFR* mutations				
Yes	3	1 (33.3)	2 (66.7)	1.00
No	26	8 (30.8)	18 (69.2)	
*P53* mutations				
Yes	8	3 (37.5)	5 (62.5)	0.67
No	21	6 (28.6)	15 (71.4)	
*PIK3CA* mutations				
Yes	4	3 (75)	1 (25)	0.076
No	25	6 (24)	19 (76)	
CDKN2A mutations				
Yes	4	0 (0)	4 (100)	0.28
No	25	9 (36)	16 (64)	
*FGFR3* mutations				
Yes	2	1 (50)	1 (50)	0.53
No	27	8 (29.6)	19 (70.4)	

### 3.6 Prognosis Analysis

**FIGURE 5:**
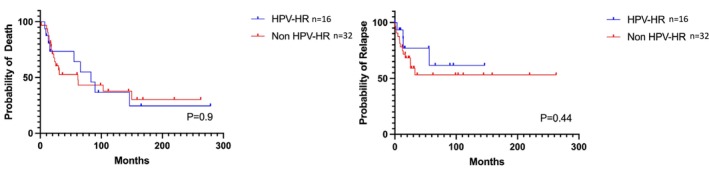
Kaplan–Meier analysis for the overall survival (probability of death) and relapse‐free survival (probability of relapse). HR‐HPV, high‐risk human papillomavirus. [Color figure can be viewed at wileyonlinelibrary.com]

We did not identify a worsening of the OS or RFS in the oncogenic HPV‐driven tumors, compared with the nononcogenic HPV‐driven tumors (Figure 5).

## 4 Discussion

Oncogenic HPV infections have been recognized as the etiological mechanism of some SNSCCs. The detection rate of oncogenic HPV in these lesions varies considerably among studies, ranging from 8.9% to 30% [9, 10, 11, 12, 13]. These discrepancies may be explained by the different techniques used to diagnose HPV‐related tumors. In the present study, we determined whether SNSCC was truly associated with transcriptionally active oncogenic HPV using the best available test methodology. Some authors judge a HNSCC to be HPV positive if it is also positive for *E6*/*E7* mRNA or p16 combined with the presence of HPV‐DNA [7, 14, 15]. Thus, to determine the rate of transcriptionally active oncogenic HPV in SNSCCs, we used IHC for p16, combined with the detection of HPV‐DNA by PCR genotyping. Discordant cases were further investigated by ISH to determine the *E6*/*E7* mRNA expression. To our knowledge, this is the first study to use this robust methodology. Our data showed that 33.3% of SNSCCs were oncogenic HPV‐driven tumors. We suggest that our results provide better insights than the recent studies conducted by Jiromaru et al. [12] and Hongo et al. [13], who found transcriptionally active oncogenic HPVs in 8.9% and 7.5% of SNSCCs, respectively. In these studies, active oncogenic HPV infection was analyzed only based on HPV‐RNA detection using ISH, and p16 overexpression was not considered while determining the HPV status. Importantly, among the cohort used in the study by Hongo et al., 10 cases were discordant, showing p16 overexpression, but were HPV‐RNA ISH negative. Falsenegative HPV‐RNA detection by ISH has been reported [25] as a false positive HPV‐DNA due to cross contamination [26]. p16 is a relatively accurate marker for oncogenic‐HPV infection within the oropharynx; however, recent reports suggested that it might be unsuitable for use in other HNSCCs localization where p16 overexpression is frequently observed, regardless of the presence of HPV [27]. Therefore, we suggest that our approach for the evaluation of oncogenic HPV‐driven tumors is robust, showing that one‐third of SNSCCs are truly oncogenic HPV‐driven tumors.

All discordant cases were p16 negative/oncogenic HPV‐DNA positive, whereas no p16‐positive/oncogenic HPV‐DNA‐negative SNSCCs were identified. Using RNA‐ISH for these discordant cases, we concluded that most patients (6/10) were p16 negative/oncogenic HPV‐DNA positive/RNA‐ISH positive. The inactivation of p16 in oncogenic HPV‐driven SNSCC tumors might be explained by CpG island methylation [28]. Interestingly, some studies have reported an increase in the methylation of the p16 promoter in lesions ranging from preneoplastic lesions to cervical cancer [29].

In oncogenic HPV‐driven OPSCC, p16 overexpression correlated with Rb loss, and some studies have suggested that a combination of p16 and Rb IHC may be a useful surrogate marker for the diagnosis of transcriptionally active oncogenic HPV in the oropharynx [20]. In the present study, the use of a combination of p16 overexpression with a complete or partial loss of Rb expression to predict active oncogenic HPV infection showed high specificity, positive predictive value, and negative predictive value (100%, 100%, and 77%, respectively), but moderate sensitivity (53.8%). This finding is not in line with the results obtained in OPSCCs, where the use of a combination of p16 overexpression with a complete or partial loss of Rb expression to predict HPV had a sensitivity of 100% [20]. The moderate sensitivity shown in our study is also lower than that observed in the SNSCC series by Jiromaru et al. (88.9%), although the specificity and positive predictive value were similar to those determined in the current study [12]. Jiromaru et al. used only ISH to detect HPV‐RNA and diagnose HPV‐positive tumors. All p16‐negative cases were oncogenic HPV‐ISH negative, and in contrast to our study, Jiromaru et al. did not conclude about the p16‐negative/oncogenic HPV‐positive cases. Based on our results, p16 overexpression alone was the most accurate marker for predicting active oncogenic HPV infection in SNSCCs, with a sensitivity of 62.5% (84.6% for Rb expression loss and 53.8% for p16 overexpression + Rb expression loss) and a specificity of 100% (71.4% for Rb expression loss and 100% for p16 overexpression + Rb expression loss). These findings are similar to those reported by Mena et al. [14]. They used the double positivity for HPV‐DNA and E6*I mRNA as a gold standard to define an oncogenic HPV‐related lesion in a cohort of oropharyngeal cancer. p16 overexpression was found to be the most accurate marker to define oncogenic HPV‐related transformation, with a sensitivity of 86.57%, as compared with Rb expression loss alone or p16 overexpression combined with Rb expression loss.

*EGFR* mutations have been reported in SNSCCs, both in de novo SCCs and ISP‐SCCs [13, 16, 17] with a frequency ranging from 14.7% to 30%, showing a higher frequency in ISP‐SCCs (92.9%) than in the de novo SCCs (6.2%) [13]. In this study, we observed *EGFR* mutations in 9.1% of the SNSCC cases, and all cases were ISP‐SCCs. In ISP‐SCCs, *EGFR* mutations were observed in 37.5% of the tumors. This result is lower than that obtained in other studies, where the prevalence of *EGFR* mutations varied from 77% to 92.7% [13, 16, 17]. It is noteworthy that in the studies carried out by Sasaki et al. and Udager et al., there were more ISP‐SCC cases than in our study (21 and 22 cases, respectively, compared with eight cases with available molecular data in our study) [16, 17]. Interestingly, in contrast to studies that concluded that *EGFR* mutations and oncogenic HPV infection in SNSCCs were mutually exclusive [13, 30], in this study, we found one case (1/3) of ISP‐SCC with *EGFR* mutations that had transcriptionally active oncogenic HPV (HPV18). This patient was NKSCC p16 negative, HPV‐DNA positive, and RNA‐ISH positive. The patient died of the disease after 9 months of follow‐up.

In the present study, the most common gene mutation was *TP53* (7/33, 21%), followed by *PIK3CA* (5/33, 15%). *TP53* mutations were present in one ISP‐SCCs in association with *EGFR* mutations and in seven de novo SCCs, either alone or in association with other gene mutations or oncogenic HPV infection. In contrast, *PIK3CA* mutations were only present in de novo SCCs, either alone or in association with other gene mutations or oncogenic HPV infection. We identified *CDKN2a* mutations in 4/33 cases (12.1%) in association with other gene mutations (4/4) and oncogenic HPV in one case. One patient had ISP‐SCCs and *EGFR* mutations, whereas another patient had de novo SCCs. *FGFR3* mutations were present in two cases (2/22, 6.1%), both of which had de novo SCCs and were HR‐HPV negative, and one case had other associated gene mutations (*PIK3CA* and *CDKN2a*). The detected gene mutations have been previously reported in SNSCCs [31]. In a series of oral SCCs (OSCCs), *Gillison* et al. also Identified *TP53*, *PIK3CA*, *CDKN2a*, and *FGFR3* mutations in HPV‐positive OSCCs [32]. Interestingly, *TP53* and *CDKN2A* mutations were found more frequently in HPV‐negative OSCCs than in HPV‐positive OSCCs, in contrast to *PIK3CA* and *FGFR3* mutations that were more frequently reported in HPV‐positive OSCCs [32]. In this study, although gene mutations were significantly less common in non‐oncogenic HPV‐driven lesions compared to oncogenic HPV‐driven lesions, no differences with regard to individual mutations were identified between the two groups.

We did not identify a worsening of the OS or RFS in the oncogenic HPV‐driven tumors, compared with the nononcogenic HPV‐driven tumors. Few studies have investigated the prognostic value of oncogenic HPV status in SNSCCs. In the studies conducted by Hongo et al., oncogenic HPV SNSCCs were significantly associated with a better prognosis [12, 13]. Nevertheless, in these studies, only a few cases of oncogenic HPV SNSCCs (11 and 9 cases, respectively) were included, compared to 16 cases in our study. Larger studies are required to estimate the prognostic value of oncogenic HPVs in SNSCCs. Interestingly, HR‐HPV‐positive OPSCC and HR‐HPV‐negative OPSCC have been separated in the 8th edition of the UICC/AJCC staging system to account for the improved prognosis observed in the former, and numerous ongoing trials are examining the potential for treatment de‐intensification or novel therapeutic regimens, such as immunotherapy, in HR‐HPV‐positive OPSCC [33].

## 5 Conclusion

This large series of SNSCCs demonstrates that approximately one‐third of these tumors are oncogenic HPV‐driven lesions associated with a nonkeratinizing morphology. In addition, we found that transcriptionally active oncogenic HPV infection in SNSCCs did not affect the survival rate. As observed in oropharyngeal cancers, p16 overexpression alone had the highest sensitivity and specificity for predicting oncogenic HPV active infection (compared with Rb expression loss alone or p16 overexpression + Rb expression loss). NGS revealed that *TP53* and *PIK3CA* mutations were the most common in SNSCCs before *EGFR* mutations. For the first time, we showed that *EGFR* mutations and active oncogenic HPV infections in SNSCCs were not mutually exclusive. Furthermore, gene mutations were significantly less common in nononcogenic HPV‐driven lesions than in oncogenic HPV‐driven lesions. Further studies are needed to analyze the prognostic value of the association between mutations and oncogenic HPV infection in SNSCCs.

## Supporting information


**Data S1.** NGS panel used.

## Data Availability

The data that support the findings of this study are available on request from the corresponding author. The data are not publicly available due to privacy or ethical restrictions.

## References

[1] T. Norlander , J. E. Frödin , C. Silfverswärd , and A. Anggård , “Decreasing Incidence of Malignant Tumors of the Paranasal Sinuses in Sweden. An Analysis of 141 Consecutive Cases at Karolinska Hospital From 1960 to 1980,” Annals of Otology, Rhinology and Laryngology 112, no. 3 (2003): 236–241, 10.1177/000348940311200308. 12656415

[2] C. Thorup , L. Sebbesen , H. Danø , et al., “Carcinoma of the Nasal Cavity and Paranasal Sinuses in Denmark 1995‐2004,” Acta Oncologica 49, no. 3 (2010): 389–394, 10.3109/02841860903428176. 20001493

[3] G. Harbo , C. Grau , T. Bundgaard , et al., “Cancer of the Nasal Cavity and Paranasal Sinuses. A Clinico‐Pathological Study of 277 Patients,” Acta Oncologica 36, no. 1 (1997): 45–50, 10.3109/02841869709100731. 9090965

[4] D. Luce , A. Leclerc , J. F. Morcet , et al., “Occupational Risk Factors for Sinonasal Cancer: A Case‐Control Study in France,” American Journal of Industrial Medicine 21, no. 2 (1992): 163–175, 10.1002/ajim.4700210206. 1536152

[5] R. B. Hayes , J. W. Kardaun , and A. de Bruyn , “Tobacco Use and Sinonasal Cancer: A Case‐Control Study,” British Journal of Cancer 56, no. 6 (1987): 843–846, 10.1038/bjc.1987.303. 3435710 PMC2002409

[6] J. Nudell , S. Chiosea , and L. D. R. Thompson , “Carcinoma Ex‐Schneiderian Papilloma (Malignant Transformation): A Clinicopathologic and Immunophenotypic Study of 20 Cases Combined With a Comprehensive Review of the Literature,” Head and Neck Pathology 8, no. 3 (2014): 269–286, 10.1007/s12105-014-0527-7. 24519376 PMC4126921

[7] X. Castellsagué , L. Alemany , M. Quer , et al., “HPV Involvement in Head and Neck Cancers: Comprehensive Assessment of Biomarkers in 3680 Patients,” Journal of the National Cancer Institute 108, no. 6 (2016): djv403, 10.1093/jnci/djv403. 26823521

[8] I. G. Bravo and M. Félez‐Sánchez , “Papillomaviruses: Viral Evolution, Cancer and Evolutionary Medicine,” Evolution, Medicine, and Public Health 2015, no. 1 (2015): 32–51, 10.1093/emph/eov003. 25634317 PMC4356112

[9] L. Alos , S. Moyano , A. Nadal , et al., “Human Papillomaviruses Are Identified in a Subgroup of Sinonasal Squamous Cell Carcinomas With Favorable Outcome,” Cancer 115, no. 12 (2009): 2701–2709, 10.1002/cncr.24309. 19365846

[10] J. A. Bishop , T. W. Guo , D. F. Smith , et al., “Human Papillomavirus‐Related Carcinomas of the Sinonasal Tract,” American Journal of Surgical Pathology 37, no. 2 (2013): 185–192, 10.1097/PAS.0b013e3182698673. 23095507 PMC3545097

[11] J. A. Bishop , X. J. Ma , H. Wang , et al., “Detection of Transcriptionally Active High‐Risk HPV in Patients With Head and Neck Squamous Cell Carcinoma as Visualized by a Novel E6/E7 mRNA In Situ Hybridization Method,” American Journal of Surgical Pathology 36, no. 12 (2012): 1874–1882, 10.1097/PAS.0b013e318265fb2b. 23060353 PMC3500437

[12] R. Jiromaru , H. Yamamoto , R. Yasumatsu , et al., “HPV‐Related Sinonasal Carcinoma: Clinicopathologic Features, Diagnostic Utility of p16 and Rb Immunohistochemistry, and EGFR Copy Number Alteration,” American Journal of Surgical Pathology 44, no. 3 (2020): 305–315, 10.1097/PAS.0000000000001410. 31743130

[13] T. Hongo , H. Yamamoto , R. Jiromaru , et al., “Clinicopathologic Significance of EGFR Mutation and HPV Infection in Sinonasal Squamous Cell Carcinoma,” American Journal of Surgical Pathology 45, no. 1 (2021): 108–118, 10.1097/PAS.0000000000001566. 32868526

[14] M. Mena , M. Taberna , S. Tous , et al., “Double Positivity for HPV‐DNA/p16ink4a Is the Biomarker With Strongest Diagnostic Accuracy and Prognostic Value for Human Papillomavirus Related Oropharyngeal Cancer Patients,” Oral Oncology 78 (2018): 137–144, 10.1016/j.oraloncology.2018.01.010. 29496041

[15] G. Pannone , V. Rodolico , A. Santoro , et al., “Evaluation of a Combined Triple Method to Detect Causative HPV in Oral and Oropharyngeal Squamous Cell Carcinomas: p16 Immunohistochemistry, Consensus PCR HPV‐DNA, and in Situ Hybridization,” Infectious Agents and Cancer 7 (2012): 4, 10.1186/1750-9378-7-4. 22376902 PMC3313884

[16] E. Sasaki , D. Nishikawa , N. Hanai , Y. Hasegawa , and Y. Yatabe , “Sinonasal Squamous Cell Carcinoma and EGFR Mutations: A Molecular Footprint of a Benign Lesion,” Histopathology 73, no. 6 (2018): 953–962, 10.1111/his.13732. 30117182

[17] A. M. Udager , J. B. McHugh , C. M. Goudsmit , et al., “Human Papillomavirus (HPV) and Somatic EGFR Mutations Are Essential, Mutually Exclusive Oncogenic Mechanisms for Inverted Sinonasal Papillomas and Associated Sinonasal Squamous Cell Carcinomas,” Annals of Oncology 29, no. 2 (2018): 466–471, 10.1093/annonc/mdx736. 29145573 PMC6248771

[18] A. M. Udager , J. B. McHugh , B. L. Betz , et al., “Activating KRAS Mutations Are Characteristic of Oncocytic Sinonasal Papilloma and Associated Sinonasal Squamous Cell Carcinoma,” Journal of Pathology 239, no. 4 (2016): 394–398, 10.1002/path.4750. 27234382

[19] R. Uchi , R. Jiromaru , R. Yasumatsu , et al., “Genomic Sequencing of Cancer‐Related Genes in Sinonasal Squamous Cell Carcinoma and Coexisting Inverted Papilloma,” Anticancer Research 41, no. 1 (2021): 71–79, 10.21873/anticanres.14752. 33419800

[20] R. Jiromaru , H. Yamamoto , R. Yasumatsu , et al., “p16 Overexpression and Rb Loss Correlate With High‐Risk HPV Infection in Oropharyngeal Squamous Cell Carcinoma,” Histopathology 79, no. 3 (2021): 358–369, 10.1111/his.14337. 33450095

[21] L. Barnes , J. Eveson , P. Reichart , and D. Sidransky , “Who Classification of Head and Neck Tumours 4th Edition,” accessed February 17, 2021, https://www.livres‐medicaux.com/who‐classification‐of‐head‐and‐neck‐tumours‐4th‐edition.html.

[22] J. S. Lewis , B. Beadle , J. A. Bishop , et al., “Human Papillomavirus Testing in Head and Neck Carcinomas: Guideline From the College of American Pathologists,” Archives of Pathology & Laboratory Medicine 142, no. 5 (2018): 559–597, 10.5858/arpa.2017-0286-CP. 29251996

[23] J. A. Vendrell , D. Grand , I. Rouquette , et al., “High‐Throughput Detection of Clinically Targetable Alterations Using Next‐Generation Sequencing,” Oncotarget 8, no. 25 (2017): 40345–40358, 10.18632/oncotarget.15875. 28404952 PMC5522202

[24] P. Blateau , E. Coyaud , E. Laurent , et al., “TERT Promoter Mutation as an Independent Prognostic Marker for Poor Prognosis MAPK Inhibitors‐Treated Melanoma,” Cancers (Basel) 12, no. 8 (2020): 2224, 10.3390/cancers12082224. 32784823 PMC7463448

[25] D. A. Kerr , K. S. Arora , K. K. Mahadevan , et al., “Performance of a Branch Chain RNA in Situ Hybridization Assay for the Detection of High‐Risk Human Papillomavirus in Head and Neck Squamous Cell Carcinoma,” American Journal of Surgical Pathology 39, no. 12 (2015): 1643–1652, 10.1097/PAS.0000000000000516. 26426378

[26] A. K. Lie , B. Risberg , B. Borge , et al., “DNA‐ Versus RNA‐Based Methods for Human Papillomavirus Detection in Cervical Neoplasia,” Gynecologic Oncology 97, no. 3 (2005): 908–915, 10.1016/j.ygyno.2005.02.026. 15943992

[27] M. Lechner , A. R. Chakravarthy , V. Walter , et al., “Frequent HPV‐Independent p16/INK4A Overexpression in Head and Neck Cancer,” Oral Oncology 83 (2018): 32–37, 10.1016/j.oraloncology.2018.06.006. 30098776

[28] S. A. Foster , D. J. Wong , M. T. Barrett , and D. A. Galloway , “Inactivation of p16 in Human Mammary Epithelial Cells by CpG Island Methylation,” Molecular and Cellular Biology 18, no. 4 (1998): 1793–1801. 9528751 10.1128/mcb.18.4.1793 PMC121409

[29] F. N. Carestiato , S. M. Amaro‐Filho , M. A. M. Moreira , and S. M. B. Cavalcanti , “Methylation of p16 ink4a Promoter Is Independent of Human Papillomavirus DNA Physical State: A Comparison Between Cervical Pre‐Neoplastic and Neoplastic Samples,” Memórias do Instituto Oswaldo Cruz 114 (2018): e180456, 10.1590/0074-02760180456. 30569945 PMC6319029

[30] M. Mehrad , E. B. Stelow , J. A. Bishop , et al., “Transcriptionally Active HPV and Targetable EGFR Mutations in Sinonasal Inverted Papilloma: An Association Between Low‐Risk HPV, Condylomatous Morphology, and Cancer Risk?,” American Journal of Surgical Pathology 44, no. 3 (2020): 340–346, 10.1097/PAS.0000000000001411. 31743131

[31] S. Viitasalo , P. R. Karhemo , J. Väänänen , et al., “Exome Sequencing Reveals Candidate Mutations Implicated in Sinonasal Carcinoma and Malignant Transformation of Sinonasal Inverted Papilloma,” Oral Oncology 124 (2022): 105663, 10.1016/j.oraloncology.2021.105663. 34915258

[32] M. L. Gillison , K. Akagi , W. Xiao , et al., “Human Papillomavirus and the Landscape of Secondary Genetic Alterations in Oral Cancers,” Genome Research 29, no. 1 (2019): 1–17, 10.1101/gr.241141.118. 30563911 PMC6314162

[33] M. Lechner , J. Liu , L. Masterson , and T. R. Fenton , “HPV‐Associated Oropharyngeal Cancer: Epidemiology, Molecular Biology and Clinical Management,” Nature Reviews. Clinical Oncology 19, no. 5 (2022): 306–327, 10.1038/s41571-022-00603-7. PMC8805140 35105976

