# Supplementary material for: Oncogenic Human Papillomaviruses Drive One‐Third of Sinonasal Squamous Cell Carcinoma and Are Not Mutually Exclusive for Gene Mutations
Source: Head Neck. 2025 Jan 25;47(6):1726–35. doi: 10.1002/hed.28084 (PMC12068539; doi:10.1002/hed.28084)
Supplement: Supplementary file 1 — Data S1. NGS panel used. [file HED-47-1726-s001.docx]

| Supplementary Table S1. NGS panel used | | |  |
| --- | --- | --- | --- |
|  |  |  |  |
|  |  | Region analyzed | |
| Gene | Reference transcripts | TruSeq Custom Amplicon gene panel | Advanta Solid Tumor gene panel |
| *AKT1* | NM_001014431 | Exon 3 | Exons 3, 4 |
| *ALK* | NM_004304 | Exons 20, 21, 22, 23, 24, 25 | Exons 20, 21, 22, 23, 24, 25, 26 |
| *BRAF* | NM_004333 | Exons 11, 15 | Exons 11, 15 |
| *CDKN2A* | NM_000077 | Exons 1, 2, 3 | Exon 2 |
| *CTNNB1* | NM_001904 | Exon 3 | Exon 3 |
| *DDR2* | NM_006182 | Exon 17 | Exon 17 |
| *EGFR* | NM_005228 | Exons 18, 19, 20, 21 | Exons 18, 19, 20, 21 |
| *ERBB2* | NM_004448 | Exons 20 | Exons 10, 12, 20 |
| *ERBB4* | NM_005235 | Exons 10, 12 | Exons 10, 12 |
| *FGFR1* | NM_023110 | Exons 12, 14 | Exons 7, 9, 12, 14 |
| *FGFR2* | NM_000141 | Exons 7, 12, 14 | Exons 7, 12, 14 |
| *FGFR3* | NM_000142 | Exons 7, 9, 14 | Exons 7, 9, 14, 16 |
| *GNA11* | NM_002067 | Exons 4, 5 | Exons 4, 5 |
| *GNAQ* | NM_002072 | Exon 5 | Exon 5 |
| *GNAS* | NM_000516 | Exons 8, 9 | Exon 8 |
| *H3F3A* | NM_002107 | Exon 2 | Exon 2 |
| *H3F3B* | NM_005324 | Exon 2 | Exon 2 |
| *HIST1H3B* | NM_003537 | Exon 1 | Exon 1 |
| *HRAS* | NM_005343 | Exons 2, 3, 4 | Exons 2, 3, 4 |
| *IDH1* | NM_005896 | Exon 4 | Exon 4 |
| *IDH2* | NM_002168 | Exon 4 | Exon 4 |
| *JAK2* | NM_004972 | Exons 12, 13, 14 | - |
| *KIT* | NM_000222 | Exons 8, 9, 11, 13, 17, 18 | Exons 8, 9, 11, 13, 14, 17, 18 |
| *KRAS* | NM_033360 | Exons 2, 3, 4 | Exons 2, 3, 4 |
| *MAP2K1* | NM_002755 | Exon 2 | Exon 2 |
| *MET* | NM_001127500 | Exons 2, 14, 15, 16, 17, 18, 19, 20, introns 13 and 14 | Exons 2, 14, 15, 16, 17, 18, 19, 20, introns 13 and 14 |
| *NRAS* | NM_002524 | Exons 2, 3, 4 | Exons 2, 3, 4 |
| *PDGFRA* | NM_006206 | Exons 12, 14, 18 | Exons 12, 14, 18 |
| *PIK3CA* | NM_006218 | Exons 10, 21 | Exons 8, 10, 21 |
| *POLE* | NM_006231 | Exons 9, 10, 11, 12, 13, 14 | - |
| *PTEN* | NM_000314 | Exons 1, 2, 3, 4, 5, 6, 7, 8, 9 | Exons 1, 2, 3, 4, 5, 6, 7, 8, 9 |
| *RAC1* | NM_018890 | Exon 2 | Exon 2 |
| *SMAD4* | NM_005359 | Exons 2, 3, 9, 10, 11, 12 | Exons 9, 10, 11, 12 |
| *STK11* | NM_000455 | Exons 1, 2, 3, 4, 5, 6, 7, 8, 9 | Exons 1, 2, 3, 4, 5, 6, 7, 8, 9 |
| *TERT* | NM_198253 | Promoter | - |
| *TP53* | NM_000546 | - | Exons 2, 3, 4, 5, 6, 7, 8, 9, 10, 11 |
